# Distributed controller clustering in software defined networks

**DOI:** 10.1371/journal.pone.0174715

**Published:** 2017-04-06

**Authors:** Ahmed Abdelaziz, Ang Tan Fong, Abdullah Gani, Usman Garba, Suleman Khan, Adnan Akhunzada, Hamid Talebian, Kim-Kwang Raymond Choo

**Affiliations:** 1 Faculty of Computer Science and Information Technology, University of Malaya, Kuala Lumpur, Malaysia; 2 School of Information Technology, Monash University, Kuala Lumpur, Malaysia; 3 Computer Science Department, Comsats Institute of Information Technology, Islamabad, Pakistan; 4 Department of Information Systems and Cyber Security, The University of Texas, at San Antonio, San Antonio, Texas, United States of America; University of Connecticut, UNITED STATES

## Abstract

Software Defined Networking (SDN) is an emerging promising paradigm for network management because of its centralized network intelligence. However, the centralized control architecture of the software-defined networks (SDNs) brings novel challenges of reliability, scalability, fault tolerance and interoperability. In this paper, we proposed a novel clustered distributed controller architecture in the real setting of SDNs. The distributed cluster implementation comprises of multiple popular SDN controllers. The proposed mechanism is evaluated using a real world network topology running on top of an emulated SDN environment. The result shows that the proposed distributed controller clustering mechanism is able to significantly reduce the average latency from 8.1% to 1.6%, the packet loss from 5.22% to 4.15%, compared to distributed controller without clustering running on HP Virtual Application Network (VAN) SDN and Open Network Operating System (ONOS) controllers respectively. Moreover, proposed method also shows reasonable CPU utilization results. Furthermore, the proposed mechanism makes possible to handle unexpected load fluctuations while maintaining a continuous network operation, even when there is a controller failure. The paper is a potential contribution stepping towards addressing the issues of reliability, scalability, fault tolerance, and inter-operability.

## 1. Introduction

Software Defined Networking (SDN) [1] is a new evolutionary concept for network architecture, which separates the control plane from the data plane. The separation helps in better management of the network with efficient handling of the network traffic on different planes of the software-defined networks (SDNs) architecture. The data plane in SDN forwards network traffic based on the control plane instructions. The SDN controller builds network intelligence by observing the data plane forwarding entities and other SDN agents. No doubt, the centralized control helps in better network management; however, it always becomes a bottleneck when it comes to exchanging large volumes of data. Moreover, due to the centralized architecture of the controller, it experiences overhead as the number of user increases. Consequently, the controller becomes an obstacle to the smooth provision of service, and if the controller itself fails, the switch that it had been managing can no longer be controlled. Moreover, the SDN controller act as a single point of failure because all the forwarding decisions are dependent directly on the controller [2]. Once the SDN controller or the switches-to-controller links fail, the entire network may collapse.

The scalability, reliability, inter-operability and fault tolerance remains a challenge in centralized network architectures[3]. However, the positive aspect of SDN is that it is centralized but highly flexible and programmable at the same time. The network programmability aspects of SDNs makes unique. Moreover, the SDNs support multiple distributed SDN controllers to be connected to a network serving as backup controllers in the time of a failure. Moreover, multiple controllers allow load sharing when a single controller is overwhelmed with numerous flow requests [4]. Furthermore, multiple controllers can reduce the latency, increase the scalability and fault tolerance, and provide availability in SDN deployment. However, the main problem with this approach is to maintain the consistency among various distributed controllers. The network applications [5] will be treated improperly by the distributed controllers because of inconsistency among the controllers concerning global view of the network states [6]. In addition, multiple controllers create controller resource management problems, including controller state distribution, data sharing, consistency, and long propagation delay among multiple controllers which limits the network convergence time as well as affects the ability of the controller to respond to the various network events in minimal time such as PACKET_IN messages.

To the best of our knowledge, this is the first effort made in clustering of the distributed controller in the SDN considering the placement of the controller and the challenges such as reliability, scalability, and fault tolerance. We aim to improve network scalability, reliability and performance by implementing a distributed controller clustering in SDNs. The proposed mechanism employs multiple commercial and prominent SDN controllers in proactive and reactive mode, whereby controllers in the clusters distribute an equal role. We carried two detailed experimentation of latency and packet loss. The emulations results show promising results. The proposed mechanism decrease long propagation delay among multiple controllers improves the network convergence time and affects the ability of the controller to respond to network events in a minimal time. Moreover, the proposed mechanism significantly reduces the packet loss with a minimum overhead of the controller CPU. The emulation results increase the overall performance of the SDNs and make possible to handle unexpected load fluctuations while maintaining a continuous network operation, even when there is a controller failure. The paper is an initial attempt towards handling the reliability, scalability, interoperability, and fault-tolerance.

The remaining of the paper is organized as follows: Section 2 gives a brief overview of the SDN architecture. Section 3 discusses related work of distributed SDN controller in terms of scalability and performance. Motivations that lead to consider distributed controller clustering is discussed in section 4 and 5. Section 6 presents the proposed distributed controller architecture. Experiment setup and system evaluation are detailed in section 5. Finally, section 6 concludes the paper.

## 2. SDN network architecture

**Fig 1:**
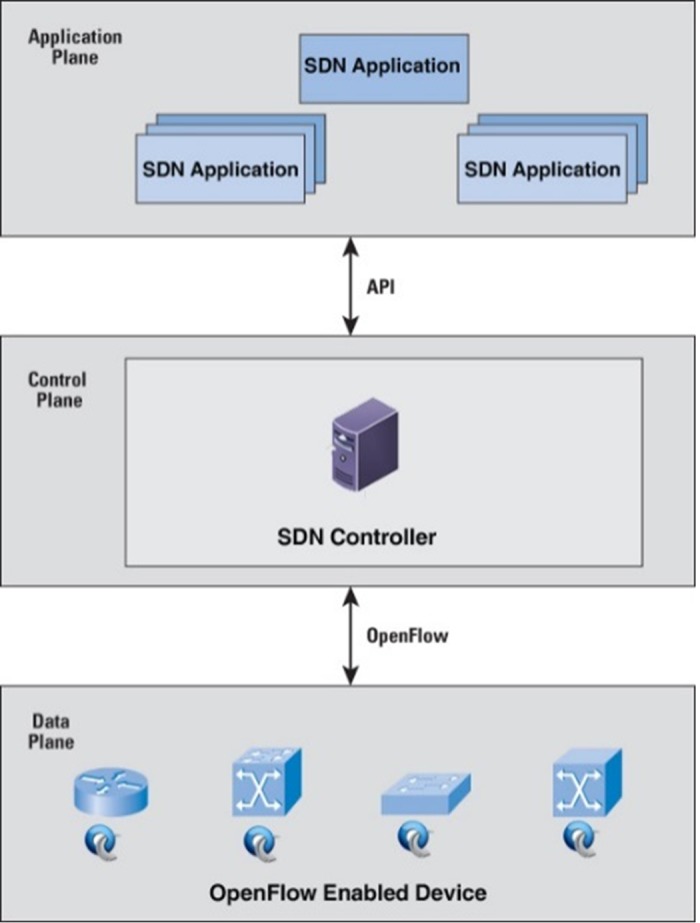
Software defined network architecture.

SDN is a new concept in computer networking, which promises to simplify network control and management and also support innovation through network programmability [7]. However, the traditional network is designed and implemented from a large number of network devices such as switches, firewalls, routers with more complex controls and protocols. The software is embedded on the network devices which require image updating whenever new features are available for its updates. Network engineers are responsible for configuring various network devices, which is a challenging and error-prone task for medium to large-scale networks. Therefore, the separation of the control plane (software) from the data plane [8] (hardware) in SDN is needed to provide more flexible, programmable, cost efficient and innovative network architecture [9]. SDN was first introduced and promoted by Open Network Foundation (ONF) to address the aforementioned issue. The SDN architecture logically centralizes the network intelligence in the software-based controllers at the control plane. The network devices (data plane)[8] simply acts packet-forwarding devices that can be programmed using an open interface called OpenFlow [10]. The separation of the control plane from the data plane enables easier deployment of new technologies and applications; network virtualization [11] and various middleboxes can be consolidated into a software control [12]. The separation of the control and data plane is compared to an operating system and the computer hardware which is illustrated in Fig 1; where the controller acts as an operating system and the forwarding devices (switches) act as the hardware devices (CPU, memory, storage). The devices are located in the south of the controller whereas network applications are located in the north of the controller. The network engineer develops customized network applications to perform various tasks such as load balancing, routing, firewall as well as traffic engineering.

## 3. Related work

Distributed controller architectures with more than one controller could be used to address some of the challenges of a single SDN controller [13] placement such as availability. In fact, a vast majority of networks contain duplication as a means to ensure the availability of the system. Furthermore, multiple controllers can reduce the latency or increase the scalability and fault tolerance of the SDN deployment. However, this architecture increases the lookup overhead of communication between switches and multiple controllers. A potential downside of this approach is to maintain the consistent state in the overall distributed system. The network applications will act incorrectly when the global view of the network state is inconsistent [6]. There has been a considerable amount of research work on distributed controller platforms such as Onix, HyperFlow, Kandoo, DISCO, Elasticon and Pratyaastha, which suggest the placement of multiple copies of SDN controllers throughout the control plane to provide scalability for larger networks and traffic loads. Onix [14] is a distributed controller for large scale networks that implements multiple SDN controllers. Onix handles the distribution and collection of information from switches and distributes controls appropriately among various controllers. A similar system with a distributed control platform is HyperFlow [15] which is an application of the NOX [16] controller that can handle state distribution between distributed controllers through a push/subscribe system based on the WheelFS [17] distributed file system. However, HyperFlow can only handle a few thousand events per second and anything beyond that is considered a scalability limitation. Kandoo [18] distributes controller states by placing the controllers in a two level hierarchy comprising a root controller and multiple local controllers. The system does not allow the controllers within a tier to communicate with one another and limit the usage of the second tier services that requiring a global network view. ElastiCon [19] proposes a controller pool, which dynamically grows or shrinks according to the traffic conditions. Besides, the workload is dynamically distributed among the controllers. Pratyaastha [20] proposes a novel approach for assigning SDN switches and partitions the SDN application state to distributed controller instances. As observed from the existing distributed controller architectures, the single point of failure of SDN controller was solved using multiple distributed controllers. However, the solution presented various challenges such as the network state distribution, the network topology consistent state, the master-selection issue and etc. As a result, this research work was carried out to address these issues.

## 4. Motivations

The common perception that the possibility for the controller to become a single-point-of-fail or a bottleneck of the network led to raising serval issues such as scalability, reliability, and performance. Numbers of research papers proposed distributed controller clustering to address these issues. In this section, we discuss these problems following by the placement of the controller in terms of distributed controller clustering.

### 4.1 Scalability

Decoupling the control plane from the data plane presents a complexity in standardizing the APIs between both planes which may lead to scalability limitations[21]. The controller becomes a bottleneck when a certain number of connected switches and end hosts, initiates more flow request than the controller can handle [22]. A study on the NOX controller has shown that the controller can handle 30K requests/sec [23]. This can be sufficient for a small to medium size network but becomes a bottleneck for a campus network or a data centre network. This is due to a large data-center network is consisting of 2 million emulated virtual switches that generate 20 million flows per second [23].

The flow-setup process increases the controller load. Besides, the network broadcast overhead and the increase of flow table entries impose limitations on the network scalability [24]. [25] proposed a distributed flow-management architecture (DIFANE), which can scale up to meet a large number of switches that generating huge flow requests. In another solution, DevoFlow [26] proposed an approach in which micro-flows are managed in the data plane and the huge flows are managed by the controller, thereby, reducing the controller load and maximizing network scalability.

### 4.2 Performance

An important performance metric of SDN is the flow-setup rate and flow-setup delay as SDN uses a flow-based technique [24]. Every flow is required to go through the controller during the flow setup process. The controller decides on the flow of traffic [27], and then, installs the flows on the switch. However, the switches are capable of generating more traffic beyond the capability of the SDN controller. For example, a controller software installed on a server over a 10 Gbps link that is in charge of switches capable of generating 1.2TB per sec of traffic [25]. Therefore, a controller may take tens of milliseconds to install a flow on the switch. In order to overcome the limitations, the factors affecting flow-setup time should be considered. Some key factors such as the processing and I/O performance of the controller were identified by [24].

### 4.3 Reliability

The SDN controller presents a single point of failure and hence the controller reduces the overall network availability in SDN [28]. In the traditional network, when there is a link failure or device failure, the network traffic is rerouted through another route or a nearby device to maintain a continuous flow of traffic. However, when a central controller fails in an SDN network, the whole network may collapse. To address this challenge, the controller is configured with a backup controller to increase network reliability [24]. Distributed controller architecture can be used to increase network reliability but the memory synchronization between multiple controllers must be maintained to avoid inconsistency in the network state [24]. The controller clustering in SDN can be used to enable continuous network availability. In the case of a controller failure, another controller in the cluster can continue to push flows to the switches, thereby providing a reliable network. The next section presents the related works on distributed controller architecture and controller placement techniques in SDN.

## 5. Controller placement problem

A study focusing on the Beacon controller [10] showed that a single SDN controller could handle 12.8 million new flows per second on a 12 cores machine, with an average latency of 24.7 ms for each flow. However, to increase scalability, reliability, robustness and fast failover, [29] recognized that the logically centralized controller must be physically distributed, as a single SDN controller architecture presents a single point of failure. Besides, the controller reliability will be affected when the switches in a network initiate more flow that the controllers can handle. A reliability-aware controller placement problem was proposed by [30] with its main objectives was to place a given number of controllers in a certain physical network such that the pre-defined objective function is optimized. The reliability issue is addressed as a placement metric that is reflected by the percentage of valid control paths. Although a tradeoff between reliability and latency shows that additional latency was incurred using the researcher’s algorithm.

In the controller placement problem, [31] developed a centralized algorithm in which a centralized controller decided the number of controllers required and the placement in the network. Although the solution is topology dependent, but, when the network grows, the solution becomes non-scalable. [32] did not address the dynamic sharing of load between the controllers in the changing network traffic instead the research was only focused on the propagation delay. [32] addressed the problem of controller placement to maximize the reliability of control networks and performed an evaluation of the trade-offs between optimizing for reliability and latency.

A fast failover for controlling traffic in SDN was presented by [33]. The authors initiated the study of controller placement for resilience and proposed a min-cut based algorithm for network partitioning and controller placement. The solution is to minimize the interruption between controller and switch links with no backup outgoing links. However, this approach cannot be applied to the environments where multiple controllers are required.

DevoFlow [26] proposed to pre-install the wildcard rules in the switches that can replicate themselves for the mice flows to create specific flow rules. The switches have the intelligence to detect elephant flows. Elephant flows are an extremely large stream of flows. Similarly, [25] developed DIFANE, in which the controller generates the forwarding rules, but, the controller is not involved in the setup of each new flow. However, both DevoFlow and DIFANE require some changes to the switches and clearly contradict the goals of SDN [34].

The impact of placing of multiple controllers in SDN was analyzed in a dynamic controller provisioning setting by [28]. Besides, the author proposed a capacitated controller placement algorithm to minimize controller load because the controller load is a critical factor in SDN-based networks. The algorithm significantly reduced the number of required controllers and the maximum load of a controller.

The location and number of the controller(s) placed in SDN play a key role in the reliability and the performance of the network. A single SDN controller presents a single point of failure. The entire SDN network will collapse in the event that the controller fails.

**Fig 2:**
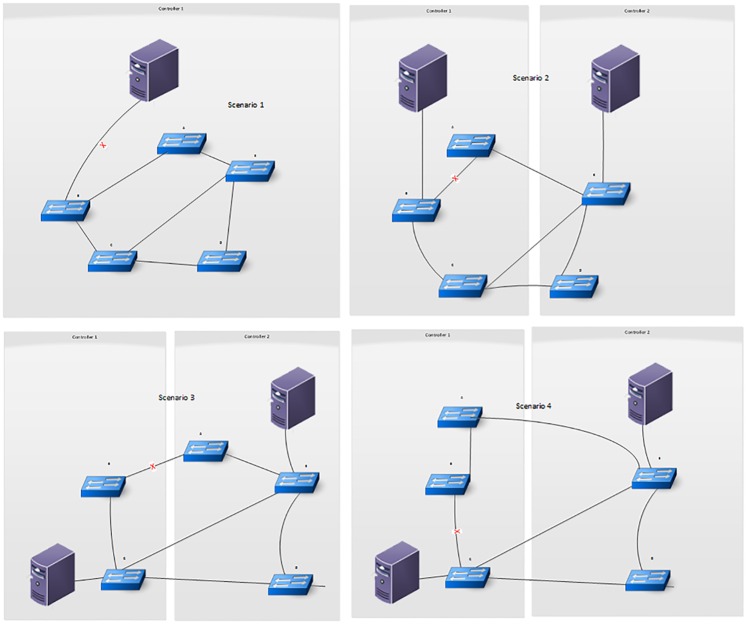
Illustrated controller placement options.

Fig 2 shows different controller placement scenarios using one or two controllers with five switches. In scenario 1, a single controller controls the switches and in the other scenarios with two controllers each, either switch can be connected to any of the controllers. The first scenario with one controller connecting the five switches is less reliable than the other scenarios because a single controller presents the single point of failure problem. The use of more than one controller also affects the reliability of the network, for instance, in the second and third scenarios where both are with two controllers but placed differently, the third scenario is more reliable than the second scenario; because when the link between switch A and switch B fails in the second scenario, the communication path between switch A and its controller is broken but when there is any link failure in the third scenario, there will be at least one communication path available, which makes it more reliable than the second scenario. The way the switches are connected to the controller also affects the reliability of the network because, in the fourth scenario, the switches are placed in exactly the same locations as the third scenario but this time switch A is controlled by controller 1 in the fourth scenario instead of controller 2. Therefore, this makes the fourth scenario less reliable than the third scenario because when there is a link failure between switch B and switch C, the communication link from both switch A and switch B to their controller which is controller 1 will be broken. As regards this observation, the controllers in the proposed system are placed using the capacitated controller placement algorithm CCPP. The (CCPP) considers the load of the controllers for its placement algorithm. The aim is to reduce the number of required controllers and analyze the load of controllers, which is mainly processing packetIn events and delivering the events to the applications. The CCPP was defined as a variant of the k-center problem and has two phases. In phase one, the lower bound of radius is obtained in binary search and in phase two, the radius is increased from lower bound until a placement is found The algorithm considers only possible distances in phase one i.e. the distance between any pair of locations rather than all integers in a given range that ensures a faster convergence. The binary search converges until the step is less than 1; it requires more iteration than searching in possible distances because the possible radius must be one of the distances, searching in possible distances will not omit the result radius. This ensures that the algorithm always finds the exact location.

## 6. Proposed architecture

**Fig 3:**
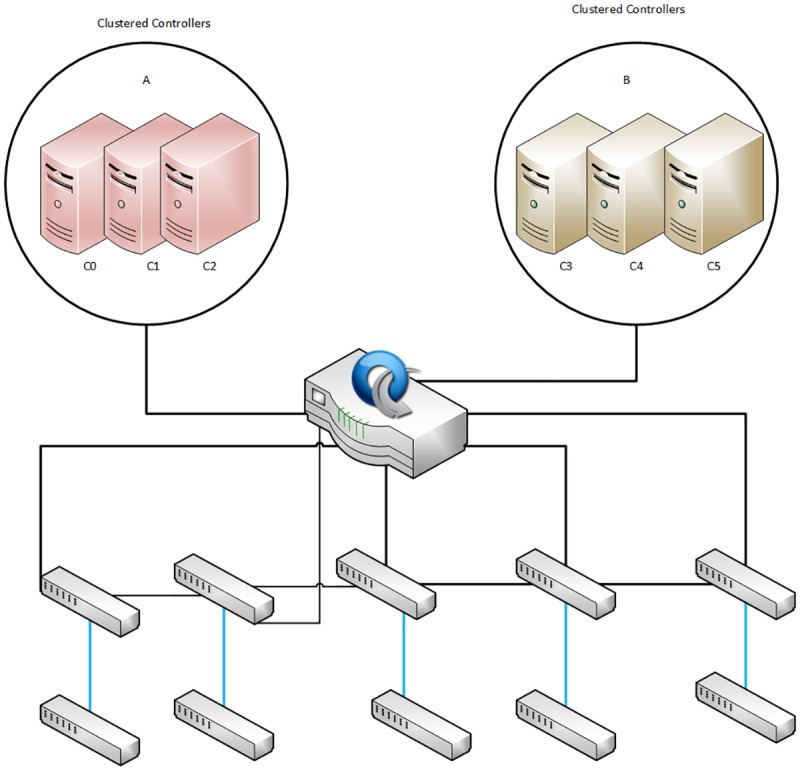
Proposed distributed controller clustering.

Our proposed architecture is based on distributed controller clustering in SDN that consists of two different types of controllers; an open source and commercial based controllers. Both types of controllers having different SDN networks. Each controller is setup within a cluster of three nodes; the controllers in the each cluster are configured in active mode with one of the controllers acting as the primary controller as shown in Fig 3. The mode provides load balancing and sharing; and network consistency among the entire cluster. In our proposed architecture, when a primary controller fails then any other controller among the cluster becomes the primary controller based on a predefined priority configuration, thus ensuring a highly available of SDN architecture. The proposed architecture is designed and implemented using ONOS and HP VAN SDN controllers configured on Amazon EC2 cloud servers. ONOS and HP VAN SDN controllers are installed and configured on different sets of three Amazon EC2 cloud servers. All servers are running Ubuntu server.

This research work presents a distributed controller clustering in SDN to address the single point of failure problem. The proposed method also uses clustering to solve the state distribution, data sharing and consistency problems of distributed SDN architectures. Fig 3 shows the architecture of the proposed system which consists of multiple controllers that are grouped in a cluster. Each controller in a cluster is a “team member” but one controller is assigned as the team manager (Primary Controller). The clustering is configured on one of the controllers and it is automatically propagated to the other controllers in the team, regardless of which controller becomes the team manager. Once the clustering configuration is completed, the team manager (Primary Controller) performs the configuration and monitoring of the controllers and their switches. If the primary controller goes down, the controller with the highest priority in the cluster becomes the team manager (Primary controller)[35]. When the failed primary controller recovers, it resumes operation only as a team member in the cluster. To configure the controllers in clusters for the proposed technique, the following requirements are considered:

- A cluster size of at least three controllers
- All controllers in the cluster must be running the same controller version
- An IP address is required for each controller
- An IP address is assigned to the cluster.

In Fig 3, the clustering A used VAN SDN controller that installed on three different Amazon EC2 servers running Ubuntu server edition 14.0 64-bit LTS. The network topology and environment are designed to meet the requirement defined by HP where there is no looping of OpenFlow switches and all the switches must be controlled by the controller. The VAN SDN controller can be installed in two modes: Standalone mode and Team mode. In the proposed method, the controllers are installed using the Team mode to provide high availability with automatic failover, resulting in a continuously managed network in the event that one controller in the team goes down. In the B clustering, the ONOS controller is installed on the Amazon EC2 Ubuntu Server 64-bit 14.0 LTS edition on three separate servers. We used the Rest API for clustering multiple controllers to share data, network consistency state and manage OpenFlow switches. The switches are connected to the master controller with the IP addresses indicating the standby controllers for each connected switch. ONOS controllers setup in an equal mode (Active–Active), the clustered controllers perform load balancing by distributing the number of connected OpenFlow switches between instances of the controllers in the cluster

## 7. Experiment setup and evaluation

In order to evaluate our proposed clustered distributed controller architecture. We conducted two experiments. The first experiment focuses on latency and the second experiment is carried out to capture the number of dropped packets (i.e. packet loss). In this section, an experimental step that includes tools and tests configuration for both experiments are detailed.

### 7.1 Network topology

**Fig 4:**
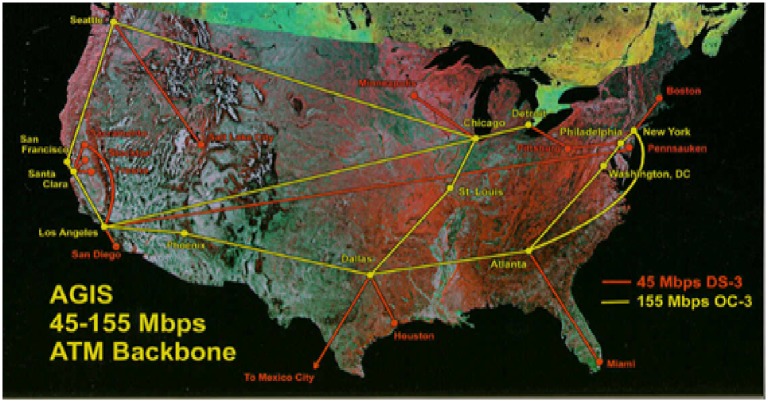
Agis network topology map.

**Fig 5:**
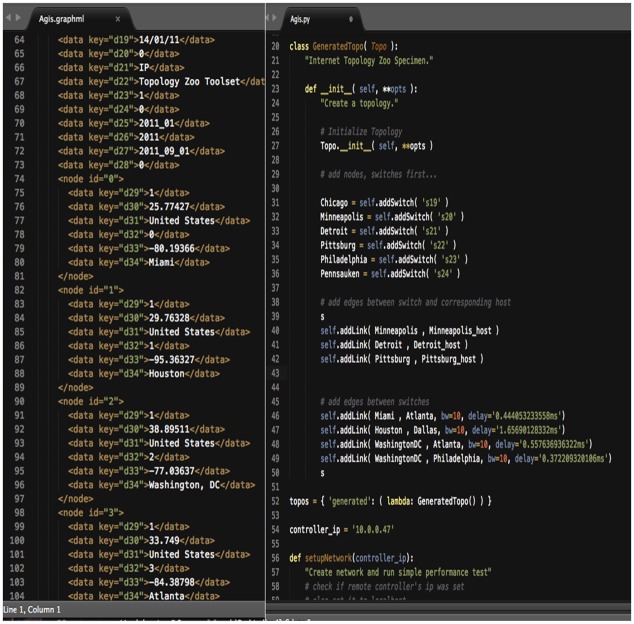
Agis topology in graphml and python script.

The research work uses a standard network topology from the Internet topology zoo (ITZ), which is a store for data of network topologies in graphical descriptions. Network operators publish information about their networks, such that the Internet topology zoo database contains topologies from AboveNet to Zamren [36]. All topologies are in a graphical format that uses the extensible markup language (XML) as description basis. The graphical format provides enough information to build up testbed networks with respect to real world topologies. Agis Network topology is used in this research work. The network contains twenty-five (25) switches, twenty-five (25) hosts with thirty (30) connected links. Fig 4 shows the Agis network topology map with all connected end points. Fig 5 depicts a section of the Agis network topology in graphml and the transformed python script of the topology. The mininet will emulate the network based on the python script of the topology.

### 7.2 Distributed Internet Traffic Generator (D-ITG)

**Fig 6:**
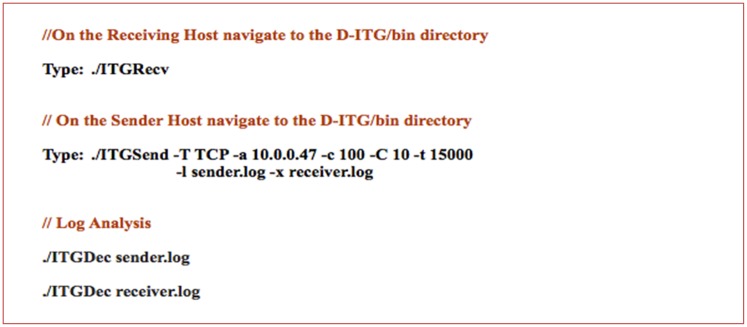
D-ITG single flow traffic.

Distributed Internet traffic generator is an application that is capable of generating traffic at the application, transport and network layers. D-ITG is used as a network measurement tool to capture the performance metrics such as delay, jitter and packet loss. Fig 6 shows the steps to generate single-flow traffic between two hosts.

In Fig 6, the ITGRecv feature of the D-ITG is run to open up a listening TCP/UDP socket for incoming traffic reception requests on the receiver. On the other hand, the ITGSend feature is run on the sender. In this example, the sender will send one TCP flow with a constant payload of 100 bytes in size and a constant packet rate of 10 packets per second for 15 seconds (15000ms).

### 7.3 Experimental setup for flow setup delay

The flow setup delay (latency) is the time taken for the controller to process a single packet. The test is carried out using Cbench. Controller bench marker (Cbench) is an application for testing SDN OpenFlow controllers by generating new flows of packetIn events towards the controller. Cbench is run in the latency mode using controller clustering of three nodes and three nodes without having to cluster. Cbench sends a PACKET_IN message to the controller and waits for the response before sending another packet. In this experiments, we used Agis Network topology, a standard network topology from the Internet topology zoo (ITZ), Tables 1 and 2 show the controller information that is used to carry out the flow setup delay (latency) test for the HP VAN SDN controller.

**Table 1:** HP VAN SDN controller information.

Controller Information	
**Controller Name**	HP VAN SDN
**Software builds version**	2.5.6
**Controllers IP Address**	10.0.0.128, 10.0.0.53 and 10.0.0.52
**Controller Operating Mode**	Distributed controllers without clustering and distributed controllers with clustering
**Connection Port**	6633
**Connection Mode**	TCP
**OpenFlow Version**	1.0

**Table 2:** ONOS controller information.

Controller Information	
**Controller Name**	ONOS
**Software builds version**	Cardinal 1.2.0
**Controller IP Address**	10.0.0.47, 10.0.0.53 and 10.0.0.52
**Controller Operating Mode**	Distributed controllers without clustering and distributed controllers with clustering
**Connection Port**	6633
**Connection Mode**	TCP
**OpenFlow Version**	1.0

Table 3 shows the test configuration metrics for both controllers. The metric used for the test is the latency and the test mode is using an incremental number of switches. The test starts with 25 switches with each switch connected to 20 hosts and subsequently, the switches are increased by 25 switches per test until maximum limit of 150 switches. The number of hosts connected to each switch remains as 20. The test duration is 10000 seconds per iteration and the total number of iteration is counted as 10.

**Table 3:** Test configuration metrics for both controllers.

Test Configuration	
**Metric**	Latency
**Number of switches**	25, 50, 75, 100, 125, 150
**Number of Hosts**	20 per switch
**Test mode**	Increment Mode
**Test Duration**	10000 (s) per iteration
**Number of Iteration**	10
**Flow measurement**	Packet_out

The controllers connect to the switches based on the real network topology from the Internet topology zoo. The controller IP address is passed to the network topology and transformed it into the python script. Afterwards, Mininet is used as the network emulator to emulate the experiment testing. In our experimental setup, we have used a real topology from the internet topology zoo. The network topology consists of twenty-five (25) switches and twenty (20) hosts with thirty (30) links. Cbench will be used as the performance tool to test the flow setup delay (latency) using a varying number of switches (25, 50, 75, 100, 125 and 100) with each switch connected to 20 hosts. We have conducted each test with a different number of iterations to have an optimal average result. The test will run for 10000 seconds, each for 10 iterations.

### 7.4 Experiment setup for packet loss

This experiment used ONOS and HP VAN SDN controllers configured on Amazon EC2 cloud servers. ONOS and HP VAN SDN controllers are installed and configured on different sets of three Amazon EC2 cloud servers. All servers are running Ubuntu server edition 14.04 LTS version. The controller will connect to the switches based on the real network topology from the Internet topology zoo. The controller IP address is passed into the network topology and transformed into the python script. Then, Mininet will be used as the network emulator to conduct the experiment testing. The packet loss test is carried out to capture the number of dropped packets during controller failover test. The test is implemented using the distributed controller architecture and the proposed controller clustering as well. The Distributed Internet traffic generator (D-ITG) is used to generate traffic. The controller failover test is carried out by streaming continuous UDP packets between two end devices using the D-ITG tool. The total numbers of packets loss are captured during the test. Table 4 shows the two controller’s setup parameters for the packet loss test. The metric that is used for the test is the packet loss in % and the test mode is using an incremental number of packets sent. The test starts with sending 1000 packets with the size of 64KB each and subsequently, increases by 1000 packets until the maximum of 5000 packets with a total size 320000 KB.

**Table 4:** Packet loss controller setup.

Controller Type	ONOS and HP VAN SDN
**Number of Cluster Nodes (CN)**	Three (3)
**Redundancy Mode (RM)**	Active–Active
**Number of Switches interconnected**	25 OpenFlow Switches
**Number of Hosts interconnected**	20 hosts
**OpenFlow Version**	1.0
**Channel Type**	TLS
**Type of Packet**	UDP
**Total Packets sent**	1000, 2000, 3000, 4000 and 5000
**Packet Size (KB)**	64

We use a standard network topology from the Internet topology zoo (ITZ), which is a store for data of network topologies in graphical descriptions. Network operators publish information about their networks, such that the Internet topology zoo database contains topologies from AboveNet to Zamren [36]. All topologies are in a graphical format that uses the extensible markup language (XML) as description basis. The graphical format provides enough information to build up testbed networks with respect to real-world topologies. Agis Network topology is used in this research work. The network contains twenty-five (25) switches, twenty-five (20) hosts with thirty (30) connected links.

## 8. Evaluation

In this section, we have evaluated our proposed architecture and discussed its output results in detail. The tests measure the latency and packet loss using the normally distributed controller architecture and the proposed controller clustering architecture in SDN. The controller flow setup delay (latency) test for HP VAN SDN and ONOS controllers are carried out to measure the time taken by the controllers to setup a flow under distributed controller architecture and the proposed controller clustering.

**Fig 7:**
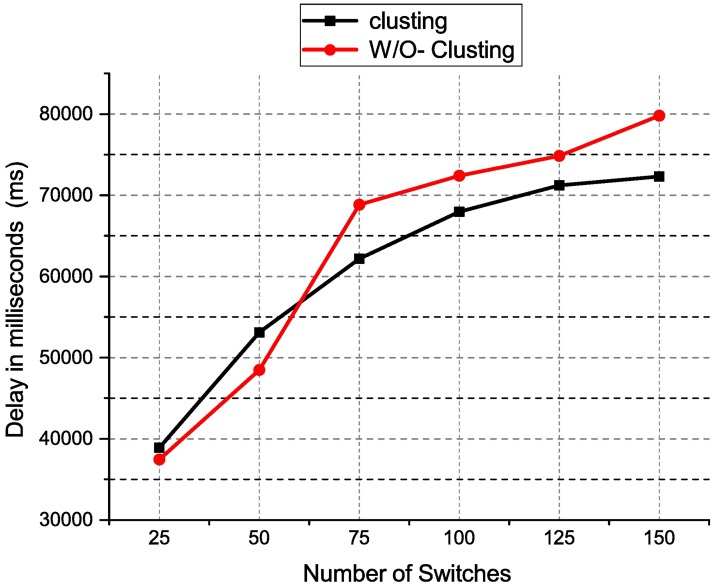
HP VAN controller latency result.

Fig 7 shows the performance chart for the HP VAN SDN latency test using distributed controller clustering and without clustering. The results show the distributed controller clustering is better than distributed controller without having to cluster. However, when the number of switches is less than 75, the latency of distributed controller clustering is higher than the distributed controller without clustering. This may be having extra synchronization overhead in distributed controller clustering. When the numbers of switches increase, the latency of distributed controller clustering is lesser as compared to distributed controller without having a clustering. The distributed controller clustering reduces the latency by an average of 8.1% when the number of switches is more than 75. This may due to clustered controllers operating as a single logical controller to the connected switches. With clustering, the processing time for packetIn messages is reduced.

**Fig 8:**
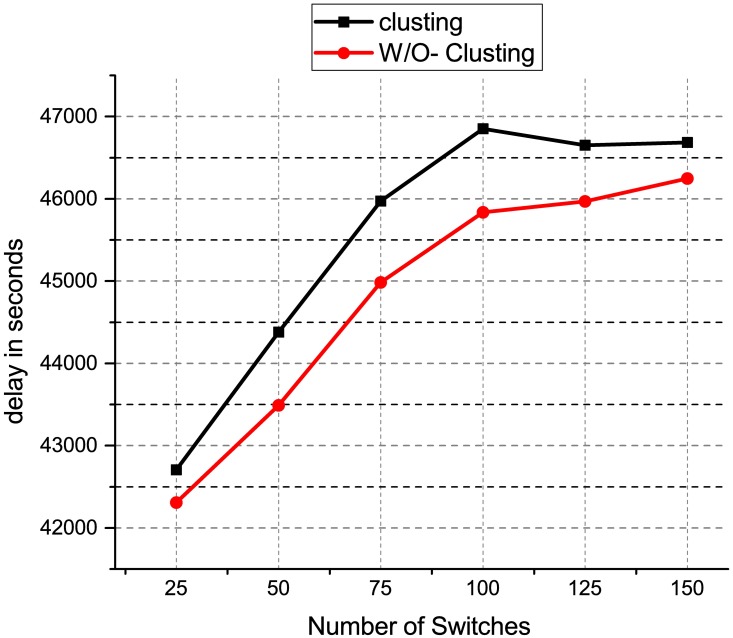
ONOS controller latency result.

**Fig 9:**
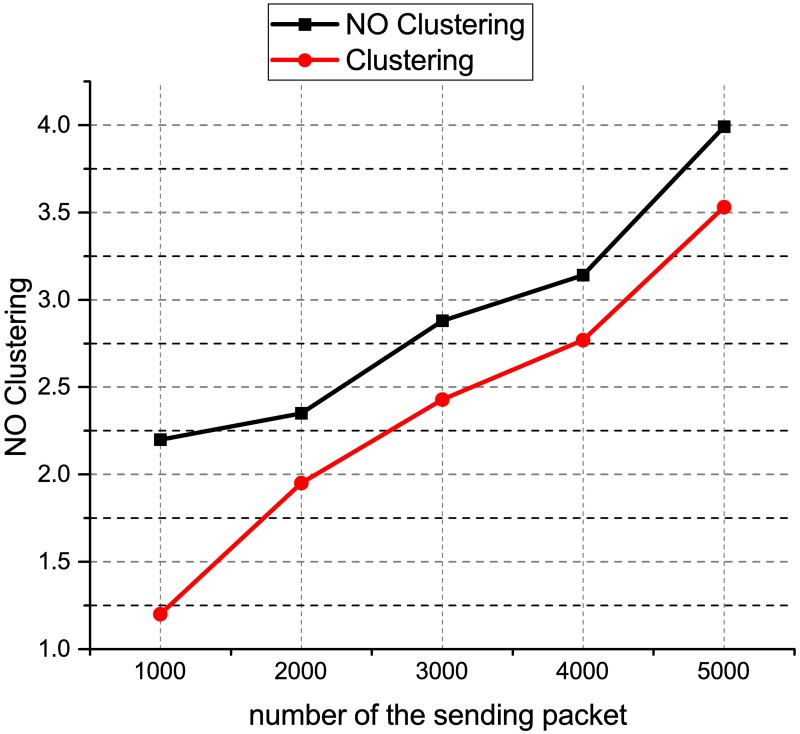
HP VAN SDN packet loss.

**Fig 10:**
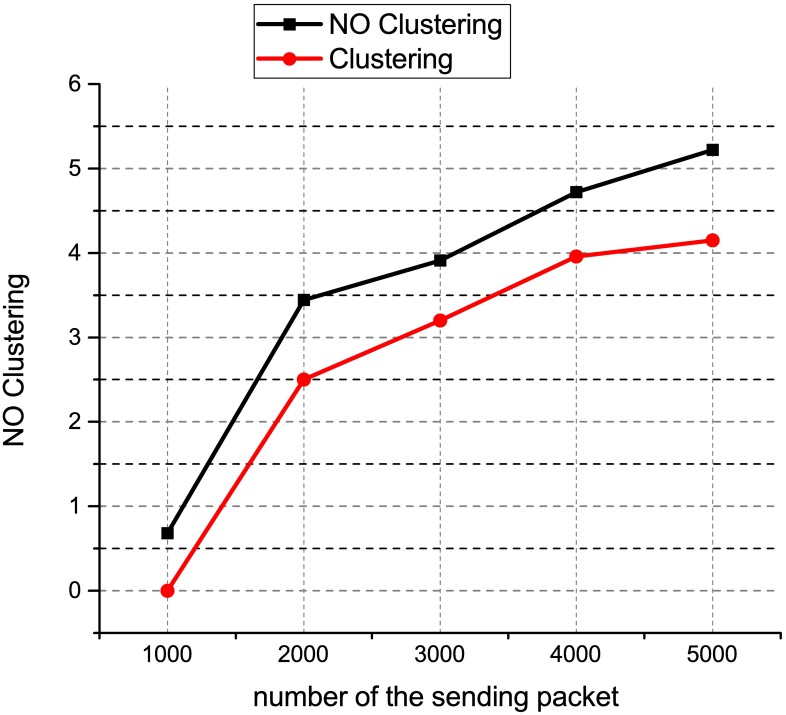
ONOS packet loss result.

Fig 8 shows the performance chart for the ONOS controller latency test using distributed controller clustering and without clustering. When the number of switches is 25, the latency is 42,309 m for distributed controller clustering as compared to 42,706 for distributed controller without clustering. Similarly, when the number of switches is 150, the latency is 46,249 ms for distributed controller clustering as compared to 46,684 for distributed controller without clustering. This result shows that the distributed controller clustering is better than distributed controller without clustering. The distributed controller clustering reduces the latency by an average of 1.6%. This may because have clustered controllers operate in a coordinated way and each controller is aware of the network state which is shared across other clustered nodes. Besides, the clustered controllers enable a load balancing function to redistribute the controller load between different clustered nodes, therefore, offering better scalability and performance as compared to the distributed controller without clustering. Fig 9 shows the results of the packet loss test for the HP VAN SDN controllers using the distributed controller clustering and without clustering. The results show that the total number of packets loss increase when the number of packets sent increase. When 5000 UDP packets with a size of 320000 KB are sent between two end devices, the percentage of packet loss for distributed controller with clustering is 3.53% as compared to 3.99% for distributed controller without clustering. The distributed controller clustering is better than distributed controller without clustering because the proposed clustering method drops fewer packets as compared to the distributed controller without clustering. This may due to the distributed controllers having difficulty in handling a coordinated control when there was a controller failure. The clustered controllers automatically reassign controllers to the switches without interruptions when a controller fails. This enables SDN-based networks to operate reliably in the event that a controller fails and reduce the number of packets loss. Fig 10 shows the results of the packet loss test for the ONOS controllers using the distributed controller clustering and without clustering. The results show that the total number of packets loss increase when the number of packets sent increase.

When 1000 UDP packets with a size of 64000 KB is sent between two end devices, no packet loss for distributed controller with clustering as compared to 0.68% for distributed controller without clustering. As the number of packets sent is increased to 5000 with a size of 320000 KB, the percentage of packet loss for distributed controller with clustering is 4.15% as compared to 5.22% for distributed controller without clustering.

**Fig 11:**
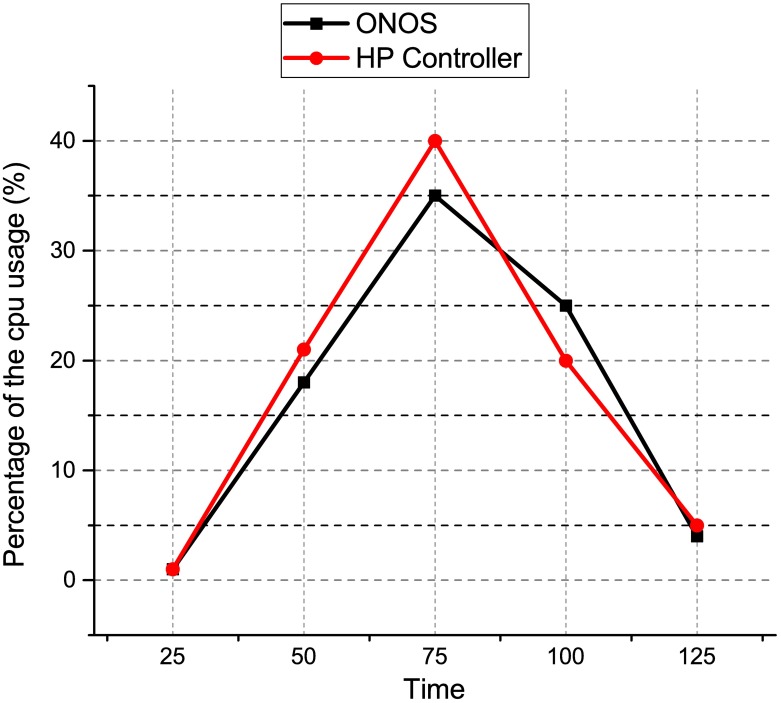
CPU utilization.

The distributed controller clustering is better than distributed controller without clustering because the proposed clustering method drops fewer packets as compared to the distributed controller without clustering. This may due to the distributed controllers have difficulty in handling a coordinated control when there was a controller failure. We further validate the fact that our proposed clustered controller architecture has negligible overheads on the controller’s performance by monitoring CPU usage for 125 seconds. We used sysbench tools to measure the CPU usage for both controllers i.e., ONOS and HP VAN controller during the clustering test. The results are presented in Fig 11, at intervals of 25 seconds. We observed that on an average, the CPU usage did not exceed 18% utilization for ONOS controller, and 21% for HP VAN controller in the normal operation. Even during the peak of activity, it does not exceed 35% and 40% utilization reactively.

## 9. Conclusion

In this paper, we propose a distributed controller clustering mechanism in SDNs. Multiple distributed prominent controllers have been configured in a cluster of three nodes in both active and reactive mode. The controller cluster is placed using the capacitated controller placement algorithm. The emulation of the proposed clustering mechanism shows promising results. The result shows that the proposed distributed controller clustering mechanism is able to significantly reduce the average latency from 8.1% to 1.6%, the packet loss from 5.22% to 4.15%, compared to distributed controller without clustering running on HP Virtual Application Network (VAN) SDN and Open Network Operating System (ONOS) controllers respectively. The result shows that the proposed distributed controller clustering outperforms the existing distributed controller without clustering in terms of latency, and packet loss with reasonable CPU utilization. In future, we consider more rigorous experimentation of diverse SDN commercial controller with different metrics such as flow setup rate (throughput), the number of nodes in the cluster and various others. Moreover, this research work can be extended to be implemented in commercial SDN-based cloud diverse data-centers infra-structures.
